# Factors Associated With Post-traumatic Stress Disorder Among Survivors of the Kamuina Nsapu Armed Conflict in the City of Kananga, Democratic Republic of the Congo

**DOI:** 10.7759/cureus.114688

**Published:** 2026-08-17

**Authors:** Joseph Tshitoko Ndawu, Adelin Nsitu Mankubu, Emmany Mbuyi Kalambayi, Ally Ndjukendi Omba, Degani Banzulu, Said Mbuku Nguala, Philippe Ntalaja Kabuayi, Grégoire Kamanga, Pascal Tshibuabua Mutshipayi, Alfred Sodi Magudigana

**Affiliations:** 1 Department of Psychiatry, Neuropsychopathologic Center, University of Kinshasa, Kinshasa, COD; 2 Department of Neuropsychiatry, Université Notre-Dame du Kasaï, Kananga, COD; 3 Department of Epidemiology, School of Public Health, University of Kinshasa, Kinshasa, COD; 4 Department of Neurology, Neuropsychopathologic Center, University of Kinshasa, Kinshasa, COD

**Keywords:** armed conflict, associated factors, insurrectionary movement, kananga, post-traumatic stress disorder

## Abstract

Background

From 2016 to 2017, the city of Kananga experienced a tragedy marked by the emergence of the Kamuina Nsapu insurrectionary movement. This armed conflict left significant human and material damage of unprecedented scale in this region of the country, where mental health service provision is extremely limited. Nine years later, the toll continues to rise. This cross-sectional analytical study was conducted to determine the factors associated with post-traumatic stress disorder (PTSD) among survivors of this armed conflict in the city of Kananga.

Methodology

Conducted from July to September 2025 using multi-stage stratified sampling, 491 respondents participated in this survey. For each participant, sociodemographic variables as well as medical, psychiatric, and traumatic histories were collected using a data collection sheet. The Post-Traumatic Stress Disorder Checklist for DSM-5 (PCL-5) was administered to screen for the disorder. Descriptive and analytical statistics were used to detail participant characteristics; logistic regression was employed to identify independent factors associated with PTSD.

Results

The prevalence of PTSD among survivors of the Kamuina Nsapu armed conflict in Kananga in 2025 was 53.4%. The median age was 40 years, with a female predominance (65.3%). Physical disability resulting from conflict (p = 0.027; OR = 5.49; CI = 1.35 - 29.58), history of cardiac conditions (p = 0.004; OR = 4.57; CI = 1.73 - 14.55), loss of valuable assets (p < 0.001; OR = 3.81; CI = 1.91 - 7.87), experiencing physical violence (p = 0.002; OR = 3.33; CI = 1.59 - 7.26), having risked death (p < 0.001; OR = 3.29; CI = 1.99 - 5.46), severe injuries of loved ones (p = 0.020; OR = 2.64; CI = 1.50 - 4.64), family psychiatric history (p < 0.001; OR = 2.48; CI = 1.52 - 4.13), death of a loved one (p = 0.001; OR = 2.34; CI = 1.15 - 4.89), and sexual violence (p = 0.001; OR = 2.34; CI = 1.17 - 4.82) emerged as independent factors statistically associated with PTSD among survivors.

Conclusion

Survivors of the Kamuina Nsapu armed conflict in the city of Kananga are particularly affected by PTSD due to the interplay of multiple factors. Tailored medico-psycho-social support remains essential in this region of the country, which is characterized by a low availability of mental healthcare services.

## Introduction

Stemming from a demand by customary chief Kamuina Nsapu in Dibaya territory, Kasai-Central province, which was inadequately managed by the provincial government, the insurrectionary movement bearing its founder's name pitted its militia against law enforcement forces from August 2016 to March 2017, causing extensive human and material damage [1,2].

Non-governmental organizations and United Nations reports note more than one million displaced persons due to this conflict, entire villages burned to ashes, and harrowing accounts from survivors revealing an overwhelming picture of multiple acts of violence and summary executions [3,4].

Among survivors, anxiety-depressive disorders and post-traumatic stress disorder (PTSD) have been reported [2], with clear repercussions on the resumption of a fulfilling socio-professional life [5].

Indeed, PTSD constitutes a serious public health issue. Its lifetime prevalence ranges from 5% to 10%, reaching higher levels among individuals exposed to highly traumatogenic events, particularly sexual violence and acts of war [6,7]. Certain groups, such as military personnel, show rates reaching 15%. It develops in 30% to 40% of victims exposed to a traumatic event, a rate that varies depending on the nature of the trauma and the victim's characteristics [6,8].

PTSD corresponds to the occurrence, following exposure to one or more traumatic events, of characteristic symptoms, namely intrusions/re-experiencing of the trauma, avoidance behaviors, negative alterations in cognitions and mood, and marked alterations in arousal and reactivity, persisting beyond one month [6].

Nine years after the Kamuina Nsapu conflict, the toll continues to worsen in Kananga. Psychologists' reports emphasize a state of disaster, with devastated communities, severed social ties, and an uncertain future [2].

Several authors subsequently focused on the socio-psychological and economic impact of this conflict in the region, such as the Regional Council of Development NGOs (CRONGD), which demonstrated how the local population's already well-known poverty was further exacerbated by this conflict [2,3].

In her study on this conflict in Kasai, Sonia Rolley highlights the unprecedented nature of both the insurrection and the subsequent crackdown, as well as the heavy toll in terms of deaths, affected localities, and related losses [4].

Given the atrocities documented during this conflict and the glaring lack of mental health services in Kananga [2], the majority of survivors did not receive appropriate medico-psychological care, leaving many of them clinically unstable [3,4]. This situation raises major concern and makes investigating the factors associated with PTSD among survivors in Kananga essential.

The objective of this study was to determine the factors associated with PTSD among survivors of the Kamuina Nsapu armed conflict in the city of Kananga, in order to contribute to improving the management of this disorder in resource-limited mental healthcare settings.

## Materials and methods

Study type and period

This was a cross-sectional, descriptive, and observational study conducted over a three-month period, from July to September 2025. The study was carried out in the city of Kananga, Central Kasai province, Democratic Republic of the Congo.

Study population

The study targeted the communes that were most impacted during the clashes. These targeted areas are located around the entrances and exits of the city, as well as those surrounding the Kananga National Airport, constituting strategic areas where clashes were most intense.

Sampling

The selection of localities and villages was carried out using multi-stage stratified sampling. First, a purposive sampling of sites was conducted based on the impact of the conflict (22 localities were selected); secondarily, avenues or clans were selected through simple random sampling. Household selection was random using a sampling interval, and within households, a single respondent meeting the inclusion criteria was selected at random.

The number of participants per village or locality was determined based on its population size. A percentage was retained on the basis of our minimum sample size.

Cochran's corrected formula was used to determine our sample size: $N = z^2 \times \frac{p\left(1 - p\right)}{m^2} \times \mathrm{DE}$

Where N = sample size, z = confidence level according to the standard normal distribution (1.96), p = estimated proportion of the population (0.5), m = margin of error tolerated (0.05), and DE = design effect (deff), used to maintain the study power given the sampling method used (cluster sampling). Taking into account accessibility, travel costs, and the sensitivity of the topic, a value of 1.5 was selected [9].

Included were women and men aged at least 18 years at the time the conflict occurred, who had been victims of at least one traumatic event linked to the Kamuina Nsapu insurrectional movement, and who freely provided informed consent.

A sample of 491 respondents was established. Sociodemographic variables, medical history, and traumatic events were recorded using a data collection form. Each respondent was administered the Posttraumatic Stress Disorder Checklist for DSM-5 (PCL-5). This is a 20-item, open-access, non-proprietary psychometric scale where a score out of 80 indicates the presence of the disorder [10].

Data processing and analysis plan

The collected data were recorded in Microsoft Excel (Microsoft Corp., Redmond, WA, USA) and exported to Stata 18 (StataCorp LLC, College Station, TX, USA) for statistical analysis. Analyses consisted of descriptive and analytical statistics. For descriptive statistics of quantitative variables, we first verified normality using the Shapiro-Wilk test under the null hypothesis. Following testing, as the p-value was below the 0.05 threshold, the quantitative variables in our database were not normally distributed. They were summarized as median and interquartile range (IQR).

As for qualitative variables, they were described using absolute frequencies and relative frequencies (percentages). We presented these variables in table or graph format.

To determine the prevalence of PTSD, we used the PCL-5 score. We then classified this score into two categories: less than 33, corresponding to the absence of PTSD, and at least 33, corresponding to the presence of PTSD.

Analytical statistics were performed to identify factors statistically associated with PTSD. To achieve this, we began by performing bivariate analyses using Pearson's chi-square test or Yates' continuity correction, depending on whether the expected frequency for each variable category was ≥ five. Variables with a p-value < 0.05 were selected for inclusion in the logistic regression model used to identify variables statistically associated with PTSD. Finally, we performed stepwise backward logistic regression.

Variables that were highly correlated with each other were identified. Age and sex, although not associated with PTSD in bivariate analysis, were retained in the final model to control for potential confounding bias, as these variables are traditionally considered major confounding factors.

Following logistic regression analysis, the discrimination and calibration of the final model were verified using the Hosmer-Lemeshow test and the area under the receiver operating characteristic curve (AUC).

Ethical considerations

Prior to the start of the study, approval was obtained from the Ethics Committee of the School of Public Health at the University of Kinshasa (Approval No.: ESP/CE/79B/2025).

Participants in whom a mental health issue was detected-specifically disabling PTSD symptoms-were referred to health facilities.

## Results

During the study, a total of 639 households were contacted. Of these, 83 households did not complete the entire questionnaire, while 65 households had no individuals meeting the eligibility criteria. A total of 491 households met the eligibility criteria and completed the entire questionnaire, resulting in a net acceptance rate of 85.54%. Of the 491 respondents, 262 tested positive for PTSD on the PCL-5, representing a prevalence of 53.4% (95% CI: 48.9-57.7). The median PCL-5 score among PTSD-positive subjects was 43.09 (IQR: 42.30-43.88).

The median age of respondents who screened positive for PTSD was 40 years (IQR: 30-51 years). Within this group, females predominated, representing 65.3% of the total. In bivariate analysis, only area of residence (p = 0.009) and religion (p = 0.004) were significantly associated with PTSD (Table 1).

**Table 1:** Percentage distribution of sociodemographic characteristics and background of respondents according to PTSD status in Kananga, 2025 PTSD: post-traumatic stress disorder; Others*: smallholder farmer, day laborer/odd-job worker, fisherman, etc; Others^†^: Jehovah's Witnesses, Sainte Sarra, Muslim, Kimbanguist, etc. Tests used for bivariate analyses: Mann-Whitney test for age, threshold α=0.05; Pearson's chi-square test for other variables, threshold α=0.05.

Characteristics	PTSD yes n=262 (53.4%)	No PTSD n=229 (46.6%)	Statistic	p-value
Age (years; median and IQR)	40 (30–51)	41 (30–56)	0.943	0.346
Sex			2.545	0.643
Female	171(65.3)	154 (67.3)		
Male	91 (34.7)	75 (32.7)		
Marital Status			1.854	0.603
Married	156 (59.6)	145 (63.3)		
Divorced	21 (8.0)	22 (9.6)		
Widowed	71 (27.1)	51 (22.3)		
Single	14 (5.3)	11 (4.8)		
Educational Level			0.732	0.866
None	13 (5.0)	11 (4.8)		
Primary	68 (26.0)	67 (29.3)		
Secondary	169 (64.5)	142 (62.0)		
Higher/University	12(4.5)	9 (3.9)		
Occupation			7.389	0.060
Small vendor/trader	78 (29.8)	48 (21.0)		
Civil servant	17 (6.5)	26 (11.3)		
Farmer	102 (38.9)	92 (40.2)		
Other*	65 (24.8)	63 (27.5)		
Place of Residence			6.890	0.009
Urban	80 (30.5)	96 (41.9)		
Urban-rural	182 (69.5)	133 (58.1)		
Religion			15.362	0.004
Revival Church	74 (28.2)	70 (30.5)		
Catholic	90(34.4)	45 (19.7)		
Protestant	21 (8.0)	28 (12.2)		
New Apostolic	22 (8.4)	19 (8.3)		
Others†	55 (21.0)	67 (26.3)		

Having a medical history, particularly a history of metabolic, cardiac, dyspeptic, and osteoarticular conditions, had an impact on respondents testing positive on the PCL-5 (p ≤ 0.001). Similarly, having a family psychiatric history, the type of previous care received, and receiving psycho-medical or traditional care were also associated with positivity on the PCL-5 (p < 0.001; Table 2). 

**Table 2:** Percentage distribution of medical and psychiatric history of respondents according to PTSD status in Kananga, 2025 Test used for bivariate analyses: chi-square test, threshold α=0.05. PTSD: post-traumatic stress disorder

Characteristics	PTSD yes n=262 (53.4%)	No PTSD n=229 (46.6%)	Statistic	p-value
Medical history				
Metabolic diseases	122 (46.6)	72 (31.4)	11.695	0.001
Cardiac conditions	49 (18.7)	5 (2.2)	34.065	<0.001
Dyspeptic syndrome	158 (60.3)	103 (45.0)	11.528	0.001
Osteoarticular conditions	176 (67.2)	104 (45.4)	23.612	<0.001
Family history of psychiatric disorders			50.749	<0.001
No psychiatric history	167 (63.7)	202 (88.2)		
History of psychiatric disorders without follow-up	76 (29.0)	22 (9.6)		
History of psychiatric disorders with follow-up	19 (7.3)	5 (2.2)		
Prior Care/Management Related to Psychotrauma				
Psycho-medical care	46 (17.6)	9 (3.9)	13.856	<0.001
Traditional care	31 (11.8)	4 (1.8)	15.909	<0.001
Any type of care	79 (30.2)	26 (11.4)	19.006	<0.001

Respondents who experienced the loss of a loved one were more represented among PTSD-positive subjects (92.5%). All stressful events, except for occasional physical injuries and lack of sharing about the trauma with others, were associated with respondent positivity (p < 0.05; Table 3). 

**Table 3:** Percentage distribution of traumatic events reported by respondents in Kananga, 2025 Test used for bivariate analyses: chi-square test, threshold α=0.05. PTSD: post-traumatic stress disorder; Other events^†^: miscarriage, impregnated by assailant, sexually transmitted infection/HIV resulting from rape during conflicts.

Characteristics	PTSD yes n=262 (53.4%)	No PTSD n=229 (46.6%)	Statistic	p-value
Death of a loved one	241 (92.0)	176 (76.9)	21.851	<0.001
Malicious destruction of property	237 (90.5)	123 (53.7)	84.347	<0.001
Having risked death	199 (76.0)	62 (27.4)	32.229	<0.001
Serious injuries to loved ones	193 (73.7)	48 (21.0)	135.812	<0.001
Torture and/or abduction	108 (41.2)	65 (28.4)	8.824	0.003
Physical violence suffered	129 (49.2)	13 (5.8)	101.806	<0.001
Sexual violence experienced	86 (32.8)	48 (21.0)	8.667	0.003
Accidental physical injuries	58 (22.1)	29 (12.7)	2.523	0.070
Loss of valuable property	240 (91.6)	119 (52.7)	93.106	<0.001
Lack of outlook for the future	195 (74.4)	111 (49.1)	79.269	<0.001
Failure to resume former professional activities	65 (24.8)	16 (7.1)	88.635	<0.001
Lack of sharing trauma with others	35 (13.4)	25 (11.1)	3.862	0.441
Physical disability resulting from the conflict	21 (8.0)	3 (1.3)	8.896	0.001
Other events†	37 (14.12)	49 (21.4)	4.477	0.034

A history of cardiac condition, physical disability resulting from the conflict, loss of valuable property, experiencing physical violence, severe injury of loved ones, having faced a life-threatening situation, death of a loved one, sexual violence, and family psychiatric history are factors statistically associated with PTSD in Kananga (Table 4). 

**Table 4:** Bivariate and multivariate analysis of risk factors associated with PTSD in Kananga, 2025 Test used for multivariate analyses: binary logistic regression reporting OR; Model discrimination and calibration were verified using the Hosmer-Lemeshow test and the area under the receiver operating characteristic curve (AUC). The model demonstrates good calibration (p=0.062>0.05) and strong discrimination (AUC=0.89), ensuring that the calculated risk probabilities faithfully reflect real-world observations in Kananga. PTSD: post-traumatic stress disorder

Characteristics	Bivariate		Multivariate		
	OR	p-value	OR	95% CI	p-value
Age	0.8	0.346	0.8	(0.67-1.03)	0.408
Sex	1.3	0.643	1.5	(0.7-8.4)	0.893
Residence	0.9	0.004	1.04	(0.8-1.3)	0.66
Religion	0.9	0.004	1.04	(0.8-1.3)	0.66
Metabolic diseases	1.2	0.001	1.9	(0.7-1.9)	0.491
Heart conditions	10.3	<0.001	4.6	(1,7-14.6)	0.004
Dyspeptic syndrome	1.8	0.001	0.3	(0.0-3.6)	0.344
Osteoarticular conditions	2.5	<0.001	1.3	(0.7-2.2)	0.389
Personal psychiatric history	2.9	<0.001	1.5	(0.9-2.4)	0.096
Family history of psychiatric disorders	3.1	<0.001	2.5	(1.5-4.1)	<0.001
Torture and/or abduction	3.9	0.001	1.89	(1.1-2.3)	0.833
Sexual violence	1.7	0.003	0.6	(0.3-1.4)	0.266
Serious injuries to relatives	1.8	0.003	2.3	(1.2-4.8)	0.001
Death of a loved one	10.5	<0.001	2.6	(1.5-4.6)	0.02
Accidental physical injuries	3.5	<0.001	2.3	(1.1-4.9)	0.019
Severe habitat destruction	8.2	<0.001	1.3	(0.4-4.5)	0.679
Having risked death	8.3	<0.001	3.3	(2.0-5.5)	<0.001
Physical violence experienced	15.9	<0.001	3.33	(1.6-7.3)	0.002
Lack of future orientation	1.2	<0.001	2.2	(0.9-4.8)	0.06
Physical disability resulting from the conflict	6.4	0.003	5.5	(1.3-29.6)	0.027
Loss of material possessions	9.8	<0.001	3.8	(1.9-7.9)	<0.001
Not returning to previous occupational activities	4.3	<0.001	1.7	(0.8-3.6)	0.188
Any type of care	0.9	<0.001	0.8	(0.3-2.1)	0.616
Psycho-medical care	5.2	<0.001	1.4	(0.5-3.7)	0.533
Traditional care	7.5	<0.001	1.4	(0.2-8.9)	0.879

## Discussion

Prevalence of PTSD in Kananga

The prevalence of PTSD in Kananga in 2025 was 53.4%. This high rate, persisting nine years after the event, highlights the atrocious and degrading nature of the conflict in the region. Over one million people were displaced, entire villages were reduced to ashes, and widespread violence occurred [4]. It should be noted that our study specifically targeted individuals who were exposed to traumatic events during the Kamuina Nsapu armed conflict.

These findings closely align with reported rates in other African studies among civilian populations. A meta-analysis indicated that PTSD prevalence ranged from 11.7% to 74.9% [11]. In Cotonou, a prevalence of 52.09% was reported among Central African migrants, while in eastern DR Congo, a prevalence of 51.9% was observed [12,13].

Sociodemographic data and PTSD

The median age of PTSD-positive respondents was 40 years (IQR: 30 - 51 years). This reflects the relative youth of the population at the time of conflict and the roles young people played during events. Our findings closely mirror data across the African continent, where median ages of 37 to 45 years have been reported [11,14].

Among PTSD-positive respondents, 65.3% were women, and 69.5% lived in semi-urban areas. These figures are consistent with literature trends. Women represent a high-risk population for PTSD due to gendered daily activities (farming, market trading) and increased exposure to vulnerabilities, including childhood sexual trauma [11,15]. Furthermore, during the conflict, adolescents and young adults in semi-urban areas were frequently drawn into militia activity and direct clashes with local government authorities.

Multivariate analysis of risk factors associated with PTSD in Kananga

Physical disability resulting from conflict (p = 0.027; OR = 5.49; CI = 1.35 - 29.58), history of cardiac conditions (p = 0.004; OR = 4.57; CI = 1.73 - 14.55), loss of valuable assets (p < 0.001; OR = 3.81; CI = 1.91 - 7.87), experienced physical violence (p = 0.002; OR = 3.33; CI = 1.59 - 7.26), having risked death (p < 0.001; OR = 3.29; CI = 1.99 - 5.46), severe injuries of loved ones (p = 0.020; OR = 2.64; CI = 1.50 - 4.64), family psychiatric history (p < 0.001; OR = 2.48; CI = 1.52 - 4.13), death of a loved one (p = 0.001; OR = 2.34; CI = 1.15 - 4.89), and sexual violence (p = 0.001; OR = 2.34; CI = 1.17 - 4.82) emerged as independent factors statistically associated with PTSD among survivors in Kananga.

This can be explained by the reality that civilian populations bear the brunt of armed conflicts without psychological preparation, suffering the sudden collapse of vital support structures (family, home, public services). Beyond direct exposure to death, survivors faced extreme cruelty, intent to destroy and humiliate, and the breakdown of social cohesion [16].

These outcomes support existing literature regarding PTSD risk factors. Across several African studies, past traumatic events; material and human losses; interpersonal violence; and female sex have been associated with PTSD risk among conflict-affected and displaced populations [13, 16, 17]. Additional reported factors include the nature and total accumulation of traumatic events, alongside individual baseline characteristics [11].

Limitations of the study

The study targeted only geographical zones considered most exposed during the Kamuina Nsapu conflict. Additionally, as a cross-sectional study relying on a self-report scale (PCL-5) for screening, there is a clear need for expanded, longitudinal population-wide studies in Kananga led by multi-professional teams using structured clinical interviews and comprehensive diagnostic tools to establish definitive clinical diagnoses.

## Conclusions

The observed results highlight that survivors of the Kamuina Nsapu armed conflict in the city of Kananga are particularly affected by PTSD. A large number of them lack appropriate support, even as their clinical condition is aggravated by the interplay of several factors, notably physical disability resulting from the conflict, medical and psychiatric history, loss of valuable property during the conflict, physical violence experienced by survivors or their relatives, sexual violence, traumatic grief, and having faced a near-death experience.

In light of these concerning findings, implementing specialized medical, psychological, and social support appears essential and urgent in this region of the country, which is characterized by a low supply of mental healthcare services.
